# Half a Century of Research on Membrane-Containing Bacteriophages: Bringing New Concepts to Modern Virology

**DOI:** 10.3390/v11010076

**Published:** 2019-01-18

**Authors:** Sari Mäntynen, Lotta-Riina Sundberg, Hanna M. Oksanen, Minna M. Poranen

**Affiliations:** 1 Center of Excellence in Biological Interactions, Department of Biological and Environmental Science and Nanoscience Center, University of Jyväskylä, FI-40014 Jyväskylä, Finland; ssmantynen@ucdavis.edu (S.M.); lotta-riina.sundberg@jyu.fi (L.-R.S.); 2 Department of Microbiology and Molecular Genetics, University of California, Davis, CA 95616, USA; 3 Molecular and Integrative Biosciences Research Programme, Faculty of Biological and Environmental Sciences, University of Helsinki, FI-00014 Helsinki, Finland

**Keywords:** *Tectiviridae*, *Cystoviridae*, *Corticoviridae*, *Plasmaviridae*, lipid-containing bacteriophage, virus–host interaction, virus evolution

## Abstract

Half a century of research on membrane-containing phages has had a major impact on virology, providing new insights into virus diversity, evolution and ecological importance. The recent revolutionary technical advances in imaging, sequencing and lipid analysis have significantly boosted the depth and volume of knowledge on these viruses. This has resulted in new concepts of virus assembly, understanding of virion stability and dynamics, and the description of novel processes for viral genome packaging and membrane-driven genome delivery to the host. The detailed analyses of such processes have given novel insights into DNA transport across the protein-rich lipid bilayer and the transformation of spherical membrane structures into tubular nanotubes, resulting in the description of unexpectedly dynamic functions of the membrane structures. Membrane-containing phages have provided a framework for understanding virus evolution. The original observation on membrane-containing bacteriophage PRD1 and human pathogenic adenovirus has been fundamental in delineating the concept of “viral lineages”, postulating that the fold of the major capsid protein can be used as an evolutionary fingerprint to trace long-distance evolutionary relationships that are unrecognizable from the primary sequences. This has brought the early evolutionary paths of certain eukaryotic, bacterial, and archaeal viruses together, and potentially enables the reorganization of the nearly immeasurable virus population (~1 × 10^31^) on Earth into a reasonably low number of groups representing different architectural principles. In addition, the research on membrane-containing phages can support the development of novel tools and strategies for human therapy and crop protection.

## 1. Introduction

Membranes composed of lipid bilayers with embedded proteins are essential for all cellular life forms. Many viruses parasitizing their host cells possess membranes either as an outer envelope or as a capsid-enclosed internal membrane. Some of the most notorious human pathogens, such as human immunodeficiency virus, influenza virus and Zika virus, have lipid components in their virions. Lipid membranes are also commonly detected in archaeal viruses [1]. However, lipids have been considered to be a rare feature among bacterial viruses (bacteriophages, phages for short), existing in less than 4% of the described isolates [2,3]. Nevertheless, the number of membrane-containing phages in culture and sequence collections has increased significantly in recent years providing new perspectives on virus diversity. Moreover, the technical advances in imaging, sequencing and lipid analysis have notably increased knowledge of these viruses. Membrane-containing phages are now recognized as common bacterial parasites in various ecological niches, thus having a larger role in the microbial ecology than previously recognized [4,5,6]. From a molecular biology perspective, these viruses provide ideal model systems to study membrane biosynthesis, structure, and function. In this review, we will summarize the state-of-the-art on membrane-containing bacteriophages. We will give an overall view of this fascinating group of bacterial viruses with an emphasis on the new results and theories.

## 2. Membrane-Containing Bacteriophages Form a Diverse Group of Biological Entities

Bacteriophages are generally acknowledged as the most abundant group of biological entities on Earth, outnumbering their bacterial hosts by at least ten-fold [7]. The first membrane-containing phage, Pseudoalteromonas phage PM2, was isolated fifty years ago, in 1968, several decades after the discovery of phages [8]. Since then, additional membrane-containing phages have been isolated throughout the world from various sources, including rotting plants, seawater, freshwater and sewage ecosystems. They infect a wide range of bacterial strains of medical, economic and environmental importance (e.g., *Escherichia coli*, *Pseudomonas syringae*, *Salmonella typhimurium* and *Bacillus anthracis*). Some of these phage isolates have been characterized more comprehensively and subsequently classified into the families *Corticoviridae*, *Cystoviridae*, *Plasmaviridae*, *Sphaerolipoviridae* and *Tectiviridae* by the International Committee on Taxonomy of Viruses. Most of the recently described membrane-containing phages fit into the present taxa, but the need for new families and genera has also been recognized. The family “Autolykiviridae”, comprising membrane-containing double-stranded (ds) DNA phages, is an example of such recently proposed taxa [6]. Flavobacterium phage FLiP represents another novel virus type that does not fit to any current taxa; the icosahedral protein capsid surrounding the internal membrane is uniquely combined in FLiP virions with a single-stranded (ss) DNA genome [9]. This discovery together with Salisaeta icosahedral phage 1 (SSIP-1), a membrane-containing phage with a genome sequence that hardly shares any similarity with those in the GenBank [10], demonstrates that membrane-containing phages are even more diverse than previously appreciated, and elucidates the importance of the isolation and characterization of new phages to gain a deeper understanding of the microbial diversity in various environmental niches.

Even though membrane-containing bacteriophages are relatively underrepresented among identified phage isolates, they form a remarkably diverse group differing in terms of virion morphologies, genome types and sequences as well as replication mechanisms. Some have highly complex virion structures with concentric protein and lipid layers, while others are merely proteinaceous lipid bags surrounding the genome. Moreover, both circular dsDNA and ssDNA genomes as well as linear dsDNA and dsRNA genomes exist (Table 1). Capsid-enclosed internal membrane-containing dsDNA phage PRD1 [11] and enveloped dsRNA phage phi6 [12] are among the best-characterized viral systems and the type members of the families *Tectiviridae* and *Cystoviridae*, respectively. Their life cycles have been comprehensively described and, consequently, the functions of membranes have also been well established for these phages.

Tailed bacteriophages, currently classified in the *Caudovirales* order, are the most abundant group of phages, outnumbering the known membrane-containing phages by over an order of magnitude. The success of the tailed virion morphology among bacterial viruses may be related to the efficiency of the tail devise in the process of bacterial cell envelope penetration. The fact that tailed virion morphologies are not known among viruses infecting wall-less eukaryotic cells supports this hypothesis. Furthermore, tailed phages with long contractile tails (myoviruses) generally have a relatively wide host range, which supports their reproduction in changing microbial communities [13]. The limited number of membrane-containing phage isolates in the previous studies could also partially be explained by their specific phenotypic characteristics that can make them subject to systematic loss in culture-based studies [6]. Firstly, membrane-containing phages are sensitive to chloroform, a reagent traditionally used in viral preparation to avoid bacterial contaminations [6,14]. Moreover, their low buoyant densities are outside the range commonly targeted when extracting phages from environmental samples using density gradient ultracentrifugation [6,15,16]. These factors should be considered when preparing samples for virus isolation and metagenomics to achieve a more complete view of the viral universe.

## 3. Phage Lipids and Their Identification

Bacteriophages do not carry inherent machinery for lipid biosynthesis, but their lipids are acquired from the host cytoplasmic membrane during virion assembly. Thus, the lipid composition of the phage reflects that of its host bacterium, at least to some extent. Bacterial membranes are primarily comprised of amphiphilic phospholipids. The most common lipid type is glycerophospholipid, in which two fatty acid side chains are linked to *sn*-glycerol-3-phosphate through ester linkages [17]. For instance, the major phospholipids of *E. coli* include zwitterionic phosphatidylethanolamine (75% of the membrane), anionic phosphatidylglycerol (20%) and cardiolipin (5%) [17].

Host-derived lipids seem to exist in phages predominantly as bilayers (membranes), which are embedded with phage-encoded proteins. For instance, the internal membrane of PRD1 is a 50:50 mixture of protein and lipid [18]. Based on the location of the membrane, phages can be roughly divided into three subclasses: (i) viruses in which the membrane is located underneath an icosahedral protein capsid; (ii) viruses in which the membrane (envelope) is the outermost layer of the virion, surrounding an icosahedral protein capsid, and; (iii) viruses in which the genome is enclosed by a proteinaceous lipid vesicle with pleomorphic appearance without any rigid protein capsid (Table 1). The lipid composition of plasmaviruses largely follows the lipid composition of its host cell membrane [19]. However, in most cases the lipids are incorporated into the virion selectively. Mechanisms leading to this selective lipid acquisition have not been established, but some possible explanations have been suggested [20]. The phage may, for instance, obtain its lipids from a membrane microdomain (lipid raft) with a distinct lipid and protein composition. On the other hand, phage-encoded membrane proteins may also selectively attract specific lipids into the virion. Moreover, certain physico-chemical properties of lipids, such as the molecular shape and charge, can drive their selective incorporation into the highly curved phage membrane, especially in the viruses with an internal membrane and in the membrane areas close to the icosahedral five-fold axes. The different phospholipids are commonly distributed asymmetrically between the phage membrane leaflets [20,21,22]. This asymmetric distribution has been suggested to reflect the different shapes and charges of the phospholipids and lipid–protein interactions [20,22].

The sensitivity to chloroform (or other organic solvents) and detergents, as well as the low buoyant density of the virion (~1.3 g cm^−3^ in CsCl) are usually the first indications of a viral lipid component [3,23]. Purified virions can also be subjected to sodium dodecyl sulfate polyacrylamide gel electrophoresis and staining with lipophilic dye Sudan Black B to detect possible lipid moieties [3]. However, these are rather indirect ways to assay the presence of lipids. For instance, chloroform can also reduce the infectivity of virions, which lack membranes [24]. Also, the density of the virion can mislead, as in case of adenovirus (1.31–1.36 g cm^−3^ in CsCl). Thus, further studies are needed to confirm the presence of lipids in virions and to determine their chemical compositions. Viral lipids are commonly extracted with organic solvents from highly purified virus particles and then subjected to thin layer chromatography, mass spectrometry (e.g., electrospray ionization) or nuclear magnetic resonance spectroscopy [25,26]. For such lipid work, highly purified viral particle material is a prerequisite to verify the presence of virus-specific lipids and to avoid misinterpreting the host-derived membrane impurities as virion components. In addition to traditional, preparative ultracentrifugation-based virus purification methods, monolithic chromatography and asymmetric flow field flow fractionation are currently available for the purification of membrane-containing phages [27,28]. Viral lipids can be directly detected from purified virus preparations by using matrix-assisted laser desorption ionization time-of-flight/mass spectrometry [3,29,30]. This technique enables the identification of lipid profiles from small amounts of viral material, without the need for a separate lipid extraction step.

The whole virus X-ray structures of phages PRD1 and PM2 (4.2 Å and 7.0 Å resolution, respectively) allowed the detailed analysis of the internal membrane and the membrane-associated proteins [22,31,32]. Today, PRD1 is still the only virus with an internal membrane solved by X-ray crystallography at high resolution, reflecting the challenges faced by the crystallization of large complexes with membrane components. However, technical advances in electron cryomicroscopy (cryo-EM) have led to “resolution-revolution” and the structural characterization of viruses at atomic resolution, which can be conducted without the need for crystallization. The single particle cryo-EM, sub-tomogram averaging and localized reconstruction methods have also provided the means to resolve non-icosahedrally organised objects in virions, such as the membrane-anchored packaging complex and membranous genome delivery tube of PRD1 [33,34] as well as the polymerase subunit and the dsRNA genome in phi6 procapsid [35] and nucleocapsid [36], respectively.

All in all, membrane-containing phages can be considered as composite systems in which nucleic acids, proteins and lipids combine in a unique way to produce infectious particles [37]. Interestingly, recent atomic force microscopic studies demonstrate that phage PRD1 is more resistant to mechanical stress compared to the majority of dsDNA icosahedral viruses that lack the membrane component [37]. It has been suggested that the coupling of a stiff and brittle protein shell and a soft and ductile membrane vesicle generates structural flexibility, which facilitates the different stages of the PRD1 life cycle, including the mechanical protection role of the extracellular virion, DNA ejection via vesicle-to-tube transformation and virion assembly (see below for details).

## 4. Significance of Membranes in Phage Life Cycle

**Figure 1:**
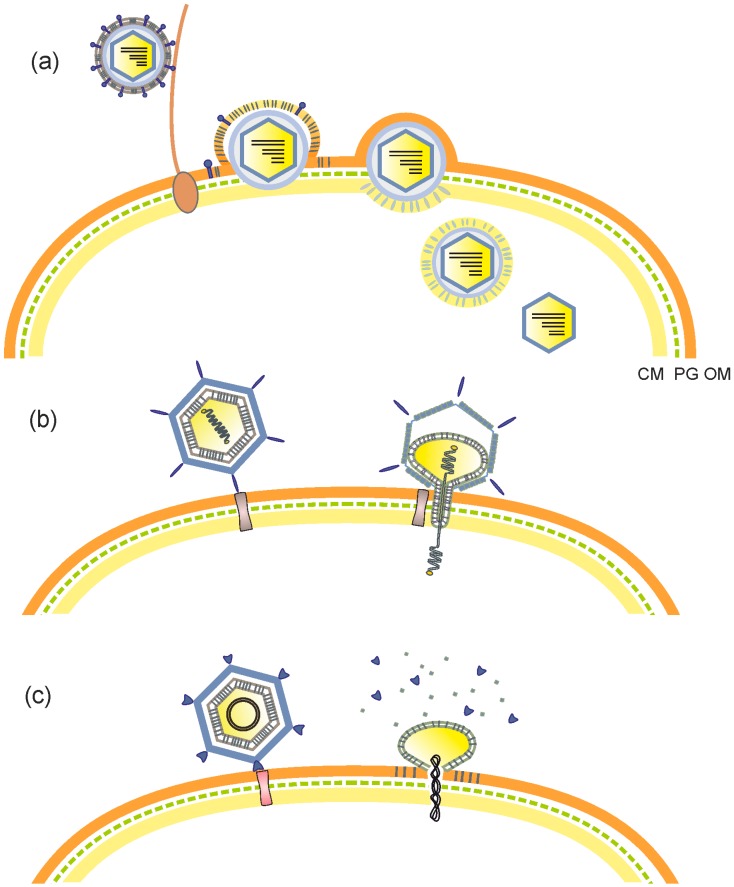
Entry mechanisms of membrane-containing phages. (**a**) Pseudomonas phage phi6. Phi6 attaches to a type IV pilus, which retracts and brings the virion into contact with the bacterial outer membrane. The viral membrane (envelope) fuses with the host outer membrane, releasing the nucleocapsid into periplasmic space. The peptidoglycan layer is digested by a virally-encoded lytic enzyme, after which the nucleocapsid enters the cytoplasm via an endocytic-like route. Finally, the nucleocapsid shell dissociates, releasing the phi6 virion core (polymerase complex). (**b**) Pseudomonas phage PRD1. Upon attachment to the host receptor, the internal membrane vesicle of phage PRD1 transforms into a proteo-lipidic tube, which traverses the cell envelope and provides a conduit for transferring the linear dsDNA genome into the cytoplasm. (**c**) Pseudoalteromonas phage PM2. It has been suggested that after phage PM2 binds to the host receptor, its protein capsid dissociates triggering the fusion between the internal membrane vesicle and the bacterial outer membrane and consequently the release of the circular dsDNA genome into the cell. CM, cytoplasmic membrane; PG, peptidoglycan layer; OM, outer membrane of the envelope in Gram-negative host bacterium.

Viruses have evolved different strategies to utilize their membranes in infection. For instance, most enveloped viruses undergo membrane fusion to deliver their genomic material into the cell [38]. Upon adsorption to a host cell, phi6, and presumably other cystoviruses, merge their envelope with the outer membrane of their Gram-negative host bacterium using phage-encoded fusion protein [39,40] (Figure 1a). The nucleocapsid surface shell, residing beneath the envelope, interacts with phospholipids of the host plasma membrane and induces the formation of cytoplasmic membrane invaginations. This process is dependent on the energized state of the host membrane and results in the delivery of the phi6 virion core into the host cell cytoplasm [41,42,43]. On the other hand, in the case of pleomorphic plasmaviruses, the fusion is assumed to take place between the virion envelope and the host cytoplasmic membrane, as *Acholeplasma* cells have no cell wall [44]. Interestingly, the capsid-enclosed internal membrane of the tectiviruses also has a dynamic role in virus entry. PRD1 and Bam35, infecting Gram-negative and Gram-positive bacteria, respectively, use their internal membrane vesicle to form a proteo-lipidic tube, which penetrates the cell envelope and provides a conduit for the passage of the linear dsDNA genome into the cell [34,45,46,47] (Figure 1b). The proteo-lipidic tube of tectiviruses forms only after receptor recognition. Binding to the receptor triggers de-capping of the vertices that leads to the loss of capsid–membrane interactions and ultimately protrusion of the tail tube, presumably from the same vertex used for packaging [34,46]. Mutations in several PRD1 membrane proteins inhibit the tube formation, suggesting their role in the membrane transformation [48]. Similarly, a proteinaceous tail structure forms and protrudes from the phage ΦX174 virion only at the time of infection and is presumably used for ssDNA genome delivery [49]. For comparison, the proteinaceous DNA injecting machinery of tailed dsDNA phages (the order *Caudovirales*) is a permanent component of the virion and energy stored in the highly dense packaged genome is one of the major driving forces in the genome injection [50]. In contrast, the packaged PRD1 genome is not required for the tube formation [34], but the internal membrane vesicle has an active role in the viral genome ejection as recently described by cryo-electron tomography [46]. A similar entry mechanism has been proposed for other phages with an internal membrane vesicle enclosing a linear dsDNA genome [34]. However, different genome types impose divergent constraints on the DNA injection, presumably giving rise to variation in the viral entry mechanisms. For instance, phage PM2 has been suggested to transfer its circular, supercoiled dsDNA genome into the host by first removing its protein capsid and then fusing its internal membrane with the outer membrane of the Gram-negative host bacterium [51,52,53] (Figure 1c).

Lipids are incorporated into the newly synthesized virions by diverse mechanisms. Most enveloped viruses obtain their external membranes as the nucleocapsids bud through the cytoplasmic membrane or one of the intracellular membrane structures, such as the endoplasmic reticulum, intermediate compartment, Golgi or *trans*-Golgi network [54]. Virus egress by budding is a delicate way to release virions without disrupting the host cytoplasmic membrane. Budding has not been confirmed for any bacteriophage but is suggested as the mode of exit for pleomorphic plasmaviruses [44]. How cystoviruses acquire their envelope is not completely understood. It has been suggested that cytoplasmic membrane vesicles are formed during the phage phi6 life cycle and that these vesicles subsequently assemble on the newly synthesized nucleocapsid to produce enveloped viruses [55]. Virus-specific vesicles can also be produced in recombinant expression systems by co-expression of the proteins involved in the envelope biosynthesis [55,56]. The mature enveloped phi6 virions are ultimately released by phage-induced host cell lysis [57]. Some variation in the assembly pathways and exit strategies of membrane-containing phages presumably results from the differences in the host bacteria: whereas plasmaviruses only need to penetrate the cytoplasmic membrane, cystoviruses also have to cross the rigid peptidoglycan layer and the outer membrane of their Gram-negative host bacteria to deliver the transcriptionally active virion core into the cytoplasm. Host-derived vesicles can also be a prerequisite for the formation of internal membrane-containing phage virions. The assembly of phage PRD1 commences as soluble capsid proteins are synthesized in the cytoplasm and phage-encoded membrane proteins are recruited into the host cytoplasmic membrane [58]. A phage-specific membrane patch, enriched in phage-encoded non-structural protein P10, is pinched off from the cytoplasmic membrane [59], in a process mimicking the clathrin-mediated endocytosis of eukaryotic cells. The resulting vesicle most probably acts as a scaffold onto which capsid proteins are assembled, ultimately displacing protein P10 and leading to the formation of an empty procapsid. Packaging ATPase P9 powers the translocation of the linear dsDNA genome into the procapsid through the unique vertex structure spanning the phage membrane [33,60,61,62,63], and finally mature virions are released by lysis. For host cell lysis, PRD1 uses a holin–endolysin system [64], in which the virion-associated muramidase P15 [65,66] and holin P35 [64,67] degrade the cytoplasmic membrane and peptidoglycan layer in timely fashion. Two additional phage-encoded lysis proteins, P36 and P37, are needed under less favorable conditions [68]. A similar assembly mechanism has been suggested for other internal membrane-containing phages with a linear genome [33]. However, the assembly of phage PM2 does not presumably occur via an empty procapsid. Instead, the encapsidation of its supercoiled dsDNA may take place concurrently with the formation of the internal membrane-containing capsid shell [32]. It has been suggested that the PM2 transmembrane proteins, P3 and P6, act as templates for the correct registration of the capsomers on the membrane. This phage-specific area of the cytoplasmic membrane interacts with the nucleic acid, leading to the pinching off of the genome-containing membrane vesicles and ultimately to liberation of mature, internal membrane-containing virions from lysed cells. The host cell lysis commences as the phage-encoded protein, P17, punctuates the cytoplasmic membrane [69]. The peptidoglycan layer is most probably disrupted by host lytic factors, whereas another phage-encoded protein, P18, helps to disintegrate the outer membrane [69].

## 5. Ecology and Evolution of Membrane-Containing Phages

Recent virus isolation and metagenomics surveys suggest that membrane-containing phages are more numerous in nature than previously thought. For instance, newly discovered internal membrane-containing phages, Toil and GC1, with unique host ranges and lifestyle features have been proposed to belong to the family *Tectiviridae* [70,71]. In addition to these fully sequenced isolates, dozens of other putative tectiviruses infecting *B. cereus* have been discovered and partially sequenced [72,73]. Also, several cystoviruses have been isolated from varying habitats in globally distant locations, demonstrating that this virus type is a common bacterial parasite in certain terrestrial habitats [4,5,74,75,76,77]. Moreover, membrane-containing dsDNA phages, “autolykiviruses”, were recently shown to be dominant parasites of ubiquitous marine bacteria of the family Vibrionaceae [6]. Furthermore, the recently found phage FLiP [9] demonstrates the potential of membrane-containing viruses in control of the abundant *Flavobacterium* hosts in freshwaters. These discoveries suggest that membrane-containing phages may play a relevant role in microbial ecology.

The assembly of membrane-containing phages is dependent on the host cell lipid pool that is utilized in a selective manner in many cases [3,23]. However, this lipid pool is not invariable as the bacterial membrane lipid composition is changing depending on ambient conditions, such as temperature, salinity, and pH [78]. Such changes in the lipid pools may influence phage–bacterium interactions in bacteria subjected to drastic environmental changes, e.g., in temperate latitudes.

Most known membrane-containing phages are virulent, but lysogenic cycles have also been described. For instance, the genus *Alphatectivirus* includes virulent phages infecting Gram-negative bacteria, whereas members of the genus *Betatectivirus* are temperate and replicate autonomously as linear plasmids in their Gram-positive host bacteria. Very recently, though, an alphaproteobacterial phage GC1 was isolated, which is the first described temperate tectivirus of Gram-negative bacteria [71]. Phage GC1 has been proposed to represent a new genus “Gammatectivirus” within the family *Tectiviridae* [71]. Lysogenic phages may have a significant role in the life cycle of their host bacteria. For instance, it has been shown that under laboratory conditions, tectiviral lysogeny has an effect on bacterial growth, the sporulation rate, biofilm formation and the swarming motility of *B. thuringiensis*, all of which are life traits involved in the survival and colonization of this bacterium in various environmental conditions [79]. Furthermore, virulent phage phi6 has been shown to be able to establish a carrier state [80], and such conditions can be boosted by introducing mutations to the gene encoding the lytic enzyme [81]. Similarly, a pseudolysogenic life cycle has been proposed for icosahedral internal membrane-containing phage KHP30 infecting *Helicobacter pylori* [82]. The frequency of carrier states or pseudolysogens in nature and consequently their role in the bacterial ecology and evolution are yet to be revealed [83]. Furthermore, although recent studies have identified several new phage resistance mechanisms [84], any possible bacterial resistance mechanisms related specifically to membrane-containing phages (e.g., restricting membrane acquisition) have not yet been identified.

At first glance, it is difficult to identify common features between different types of membrane-containing phages, which appear to be of polyphyletic origin [23]. However, high resolution structural data have provided surprising insights into the evolutionary trajectories of these viruses. Viruses can be classified into structure-based viral lineages based on common virion architectures and major capsid protein (MCP) folds [32,85,86,87,88,89]. The viral membrane components are not utilized as criteria as the presence of the membrane is not directly linked to the capsid protein fold. For now, there are four lineages, one of which is the so-called PRD1-adenovirus lineage. In fact, the concept of structure-based lineages was established after the discovery of the similar topologies of the MCPs of bacteriophage PRD1 and human adenovirus [85]. The MCPs of these viruses contain two concatenated upright β-barrels (double jellyrolls; DJR), which are stacked in a specific manner [85]. In addition to the MCP fold, PRD1 and adenovirus share the same overall virion architecture [triangulation number (*T*) = 25 icosahedral lattice] [90,91], vertex structure [31,92,93,94] and protein-primed replication mechanism. These similarities strongly suggest that PRD1 and adenovirus descended from a common ancestor. Later, similar DJR MCPs have been found in a number of viruses, including phage PM2 [32], archaeal Sulfolobus turreted icosahedral virus [95] (STIV) and Paramecium bulsaria Chlorella virus 1 (PBCV-1) infecting green algae [96]. Interestingly, the capsid of Thermus phage P23-77 is formed of two major protein types instead of one, both of which have a core fold of a single β-barrel, resembling the ones forming the DJR MCPs found in the PRD1-adenovirus lineage [97]. It has been suggested that P23-77 forms the earliest branch in the lineage. Similarly, phage SSIP-1 [10] and Thermus phage IN93 [98] display PRD1-adenovirus type architecture and encode two MCP types. P23-77, SSIP-1 and IN93 were all found from extreme environments. Most recently, the DJR fold was detected in the MCP of the phage FLiP, placing it into the PRD1-adenovirus lineage, even though its genome is ssDNA instead of dsDNA as in other members of the lineage [9]. Moreover, the DJR element has been identified in the genomes of a number of major bacterial and archaeal phyla, as well as in marine water column and sediment metagenomes, suggesting that the ecological and evolutionary importance of these viruses for microbial systems is greater than previously recognized [6,99]. The fact that the viral DJR MCP fold spans all three domains of life suggests that the lineage originated prior to the diversification of cellular life [87,100]. On the other hand, the similarities may also reflect the widespread horizontal transfer of MCP genes between viruses. Interestingly, the structure-based classification of the PRD1-adenovirus lineage is strongly supported by recently detected nucleotide and amino acid sequence similarities, further suggesting that the similarities between the lineage members result from common descent [101].

Also, cystoviruses share a common virion architecture and lifestyle with eukaryotic dsRNA viruses. These similarities reflect the common challenges faced by dsRNA viruses. Firstly, host cells lack the machinery to replicate dsRNA, and secondly, dsRNA molecules typically elicit strong cellular defense responses [89]. To overcome these challenges, the dsRNA genome is delivered into the host within a conserved core structure, the polymerase complex (PC), carrying all the enzymatic functions needed for viral replication and transcription. The phi6 PC consists of 120 copies of the MCP arranged in 60 asymmetric dimers [*T* = 1] [35,102,103]. This unique inner shell architecture has been identified in other cystoviruses [104,105], as well as in eukaryotic dsRNA viruses belonging to the families *Reoviridae* [106,107,108,109], *Picobirnaviridae* [110], *Totiviridae* [111,112,113], *Partitiviridae* [114,115], *Megabirnaviridae* [116] and *Quadriviridae* [117]. Also, the viral RNA-dependent RNA polymerases, the key enzymatic components of the dsRNA virus polymerase complexes, share a common structural fold [118].

Whereas the virion architecture is highly conserved, viral structures and functions related to host interaction are in a constant state of change, providing the means to adapt to new environments and hosts [86,87,89]. Corticovirus PM2 and the newly described corticovirus Cr39582 provide an example of such evolution with their syntenous genomes but non-homologous spike protein encoding genes [119]. Similarly, genome replication proteins may differ among closely related viruses, as in the case of aquatic PM2-like viruses [120]. Moreover, the additional virion layer present in some dsRNA viruses facilitates virus–host interaction and, therefore, displays more variability [89,121]. For instance, the sequences of the internal virion proteins and the enzymes of cystoviruses are highly conserved, but more variance is observed in proteins exposed on the virion surface (envelope proteins), resulting in different host specificities [75].

The early evolutionary history of some of the membrane-containing viruses might be shared with plasmids, offering a possible explanation for the origin of these viruses. This idea is supported by the recent finding of an Antarctic “infectious” plasmid vesicle that uses a virus-like transfer mechanism [122]. This system resembles notably pleomorphic prokaryotic viruses infecting bacterial and archaeal hosts [123,124,125].

## 6. Applications of Membrane-Containing Phages

Membrane-containing phages infect a wide range of bacterial species, many of which are pathogenic for plants, animals and humans. Thus, these viruses are potential biocontrol agents. For instance, cystoviruses have been suggested to provide means to treat bacterial infections afflicting both agriculturally important plants and humans [76]. Furthermore, conjugative plasmid-dependent phages, e.g., PRD1, provide a potential tool to restrict the horizontal spread of antibiotic resistance genes. These phages use receptors encoded by conjugative plasmids that belong to incompatibility groups IncP, IncN, or IncW and often carry antibiotic resistance genes [11], making antibiotic-resistant bacteria with conjugative plasmids targets for selective infection and removal [126,127].

Phage phi6 has provided several applications for the life sciences. The in vivo and in vitro dsRNA replication systems based on phi6 polymerase complex or RNA-dependent RNA polymerase enable the large-scale production of high-quality dsRNA molecules [81,128] to be used in RNA interference applications against plant and human pathogens [81,129,130,131,132,133] or to induce innate immunity responses in human cell lines [134]. In addition, the phi6 major membrane protein has been used to improve the correct folding of heterologous membrane proteins in bacterial expression systems [135,136] and to produce phi6-specific synthetic lipid scaffolds for enzymatic reactions in *E. coli* [137].

The discovery of new viruses from various environmental niches, from moderate to extreme, has also revealed new potential applications. Tectivirus-like phage Toil provides a novel environmentally friendly method to extract triacylglycerol from oleaginous bacterium *Rhodococcus opacus* for biodiesel production [70]. Extremophilic phages, exemplified by P23-77 from alkaline hot spring and SSIP-1 originating from extreme salinity, may also constitute an important source of novel enzymes for biotechnological applications [10,97]. In addition, the structure of P23-77 suggested mechanisms for stabilization and the assembly of protein-lipid systems at extremely high temperatures [97,138]. In general, an understanding of the mechanistic properties of the membrane-containing virus particles provides interesting new insights into the engineering of tough composite nanomaterials that are well suited to protect fragile cargos [37].

## 7. Concluding Remarks

Membranes provide ancillary means for viruses to overcome the surface barriers of the host bacteria [23]. The structures and specific functions of lipid bilayers differ greatly among phages, reflecting the enormous diversity among viruses and their remarkable ability to adapt to infect different host cells.

Recent evidence suggests that membrane-containing phages are more prevalent than has been previously thought, and they infect members of major bacterial taxa (such as *Pseudomonas*, *Bacillus*, *Vibrio* and *Flavobacterium*). Although the number of membrane-containing phage isolates is still rather limited, the genetic, structural and functional data that originate from these biological entities have been instrumental in the development of virus research, providing new insights into, e.g., virus assembly, virion structure, and infection mechanisms as well as bringing up the concept of virus lineages to group viruses based on their conserved structural elements. It is likely that future research will provide additional interesting molecular insights into the infection processes of different membrane-containing phages. Such information forms the foundation for a deeper understanding of this fascinating group of viruses, paving the way for new innovations and applications.

## Figures and Tables

**Table 1:** Virus isolates of the membrane-containing bacteriophages with a complete genome sequence.

**Schematic Representation (Not on Scale)**	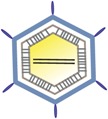	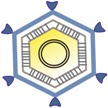	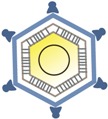	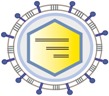	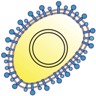
**Morphology**	Icosahedral, internal membrane	Icosahedral, internal membrane	Icosahedral, internal membrane	Enveloped	Pleomorphic
**Virion diameter**	~55–80 nm	~55–130 nm	~60 nm	~50–85 nm	~80 nm
**Genome**	dsDNA, linear	dsDNA, circular	ssDNA, circular	dsRNA, segmented	dsDNA, circular
**Life cycle**	Lytic Lysogenic	Lytic Lysogenic	Lytic	Lytic	Lysogenic ^3^
**Family**	*Tectiviridae* Unassigned	*Corticoviridae* *Sphaerolipoviridae* Unassigned	Unassigned	*Cystoviridae*	*Plasmaviridae*
**Virus isolates**	Pseudomonas phage PRD1 and PR4; Enterobacteria phage PR3, PR5, L17, and PR722; Thermus phage P37-14; Bacillus phage Bam35, AP50, phiNS11, GIL01, GIL16, and Wip1; Gluconobacter phage GC1; Rhodococcus phage Toil	Pseudoalteromonas phage PM2 and Cr39582; Thermus phage P23-77 and IN93; Salisaeta phageSSIP-1; Helicobacter pylori phage KHP30 ^1^	Flavobacterium phage FLiP	Pseudomonas phage phi6 ^2^, phi8, phi12, phi13, phi2954, phiNN, and phiYY	Acholeplasma phage L2 ^3^

^1^ Phage KHP30 may conduct a pseudolysogenic life cycle; ^2^ Phage phi6 is able to form a carrier state; ^3^ Phage L2 virions appear to be released by budding through the cell membrane, without lysing the host cell.
